# "Me's me and you's you": Exploring patients' perspectives of single patient (n-of-1) trials in the UK

**DOI:** 10.1186/1745-6215-8-10

**Published:** 2007-03-19

**Authors:** Sara T Brookes, Lucy Biddle, Charlotte Paterson, Gillian Woolhead, Paul Dieppe

**Affiliations:** 1 Department of Social Medicine, University of Bristol, UK; 2 MRC Health Services Research Collaboration, Department of Social Medicine, University of Bristol, UK

## Abstract

**Background:**

The n-of-1 trial offers a more methodologically sound approach to determining optimum treatment for an individual patient than "trials of therapy" routinely conducted in clinical practice. However, such methodology is rarely used in the UK. This pilot study explores the acceptability of n-of-1 trials to patients in the UK.

**Methods:**

Patients with osteoarthritis of the knee were recruited to their own 12-week n-of-1 trial comparing either two knee supports or an NSAID with simple analgesic. Patients were interviewed at the start and completion of their trial to explore reasons for participation, understanding of the trial design and experiences of participation. Daily diaries were completed to inform future treatment.

**Results:**

Nine patients participated (5 supports, 4 drugs). Patients were keen to participate, believing that the trial may lead to personal gains such as improved symptom control and quality of life. However, recruitment to the pharmacological comparison was more difficult since this could also entail risk. All patients were eager to complete the trial, even when difficulties were encountered. Completing the daily diary provided some patients with greater insight into their condition, which allowed them to improve their self-management. The n-of-1 trial design was viewed as a 'logical' design offering an efficient method of reaching a personalised treatment decision tailored to suit individual needs and preferences.

**Conclusion:**

This pilot study suggests that patients perceive the n-of-1 trial as an acceptable approach to the individualisation of treatment. In addition, further benefits over and above any gained from the interventions can be derived from involvement in such a study.

## Background

In routine clinical practice, when optimum treatment for an individual patient is uncertain, clinicians frequently conduct a "trial of therapy", in which the patient is given a treatment and the subsequent clinical course determines whether the treatment is deemed effective and continued. Many factors may mislead the clinician, such as the placebo effect, the natural history of the illness, patient and clinician preferences and the urge of the patient and clinician not to disappoint each other[1]. The single patient, or n-of-1 trial, offers a more methodologically sound approach to identify responders and non-responders to treatment amongst those with chronic and stable conditions and to determine optimum therapy for the individual [1-3]. Indeed, it has been suggested that the n-of-1 trial is the study design with the potential to deliver the highest strength of evidence for making individual treatment decisions[4]. In such a trial, an individual serves as their own control in assessing the comparative effectiveness of different treatments. This design provides the opportunity to measure the symptoms that matter to the individual concerned. It is conducted as a randomised, multi-crossover trial with three or more periods receiving each treatment. Ideally, the patient and health care providers are blind to the allocation of treatment within each period.

Previous research has demonstrated that n-of-1 trials can provide a definitive clinical answer as to future treatment [5-10]. Nevertheless, they are not widespread within healthcare and the design is said to be under-exploited[11]. This is especially the case in the UK. Before advocating the increased use of such methodology in clinical practice in the UK it is essential to assess its acceptability to patients. Whilst n-of-1 trial methods and examples of their use in different clinical areas[5-10,12], are reasonably well documented[1-3,10], little work has been published exploring the patient's perspective of being involved in such a trial [5-7]. N-of-1 trials rely on co-operation between individual health care providers and patients and there is a suggestion that where such trials fail it is likely to be related to non-compliance or poor symptom reporting by patients[1]. The time commitment for patients and health professionals is considerable and may lead to particular problems with recruitment and retention. The individual's commitment to the trial is therefore essential.

This paper reports a pilot study that begins to explore patients' perspectives of being involved in their own n-of-1 trial in the UK. Individual trials were conducted with patients with osteoarthritis (OA) of the knee, comparing (1) a standard knee support with a heat retaining support, or (2) a non-steroidal anti-inflammatory drug (NSAID) (diclofenac) with a simple analgesic (paracetamol).

## Methods

### Recruitment of patients

Patients attending clinics in the North Bristol Health Care Trust, with confirmed OA of the knee (Kellgren-Lawrence radiographic score of 2–4 within the previous 12 months)[13] and use-related pain were eligible. Patients who had received corticosteroid injections or operations on their knee in the previous six months were excluded. Those with known contraindications to paracetamol or any NSAID, and those taking steroids, warfarin or aspirin for another medical condition were excluded from the drug comparisons. The study was approved by the local medical research ethics committee.

The intention was to recruit around ten patients to their own 12-week n-of-1 trial, five comparing two knee supports and five comparing two drugs. The research nurse initially approached patients via telephone. A letter and information sheet explaining the study was then sent. Patients willing to participate attended an appointment with the research nurse during which the study was discussed in more detail and informed consent was obtained. Eligible patients not wishing to participate were asked if they would talk to a researcher about reasons for non-participation.

### Interventions

Each trial had a total of six treatment periods, three on each type of intervention considered. The sequence of treatments was determined by an independent researcher using computer-generated random numbers and the treatment allocation schedules were kept in consecutive sealed opaque envelopes held by the research nurse (support trials) or the dispensing pharmacy (drug trials). In the knee support trials, each treatment period was one-week long with a one-week 'washout' period between each support. It was not possible to blind the patient or the research nurse since the supports could not be made indistinguishable due to the thickness and material of the heat retaining support. During these trials, patients continued with their usual medication. In the drug trials, each treatment period lasted two weeks with no 'washout' period. Identical placebos were obtained for the diclofenac and the paracetamol. In any treatment period patients received one bottle of an active drug and one of placebo. Patients, research nurse and consultant were blind to which active drug was being taken. Patients were requested to take two 500 mg tablets of paracetamol or matching placebo three times a day and one 50 mg tablet of diclofenac or matching placebo three times a day. Patients stopped taking all currently prescribed medication for their OA for the duration of their drug trial but emergency analgesic was available. The research nurse met with patients at the end of each treatment period to give them the intervention for the subsequent period.

### Patient interviews

All participating patients were interviewed at the start of their individual trial and again on completion or termination. Interviews were qualitative and semi-structured using open-ended questioning and a topic list. The list was developed by the research team but updated throughout the study to incorporate emerging themes. The main topics explored were: decision-making surrounding participation; understanding of the n-of-1 trial design; expectations prior to commencing; and experiences of participation. Respondents were able to discuss these issues in any order, in their own terms, and to introduce other issues of importance. Interviews lasted between 45 and 90 minutes and were conducted by CP (supports trials) and LB (drug trials) at the patient's home or at hospital according to the patient's preference.

Interviews were tape-recorded and transcribed verbatim. All data were anonymised. Transcripts were read carefully and then coded to identify common themes. Codings were compared across transcripts and a descriptive coding framework was devised, which was applied systematically to all transcripts. This identified key categories of codes. Descriptive accounts were then produced to explore the content of each category and to compare and contrast this within and across interviewees, thus following the general method of constant comparison[14]. Summary case studies were also produced for each interviewee. In order to check consistency of interpretation a sample of transcripts were coded by two researchers. Data collection and analysis were alternated. Baseline interviews informed follow-up interviews and findings from knee support trial interviews were explored further with those participating in drug trials.

### Daily diaries

Patients completed a daily diary throughout their trial. This incorporated visual analogue scales for overall pain and stiffness in the study knee, a patient generated outcome measure (MYMOP) [15], and the standard disease-specific Western-Ontario and McMaster University scale (WOMAC)[16]. On completion of each trial, diary data were analysed and a summary was prepared for discussion between the patient and consultant to inform future treatment. These analyses are not presented here since this paper focuses on the interview data.

## Results

### Patient recruitment

At the end of 2001, patients were approached to participate in their own knee support trials. The first five eligible patients agreed to participate. Patients were invited to participate in the drug trials in 2003. Of the first 10 eligible patients, four gave their consent to participate and six refused: one patient was caring for their partner and was concerned about the time involved and becoming ill (this patient, Mary, agreed to talk to a researcher about her reasons for non-participation); one had participated in the knee support trials but was not keen to be involved in a trial of drugs; one was recommended by their GP not to participate due to co-morbidities; one was not interested in research; two gave no reason for refusing. Three patients withdrew before the end of their trial due to intolerance to the interventions (Table 1).

**Table 1:** Patient characteristics and trial progress

**Name^a^**	**Age**	**Regular medication prior to trial**	**Trial completion**	**Result of trial**	**Treatment decision**
*Standard vs heat retaining knee support – trials commencing December 2001 to February 2002*					
Ann	67	solpadol, meloxicam, amitryptiline	Completed	Heat retaining support beneficial	Continued use of heat retaining support
Mel	54	dihydrocodeine, co-dydramol, amitryptiline	Withdrew after 6 weeks – intolerance to supports		No further use of supports
Amber	49	no regular medication, occasional paracetamol	Completed	Heat retaining support beneficial	Continued use of heat retaining support
Lyn	54	rofecoxib, dihydrocodeine, paracetamol	Completed	No benefit from either support	No further use of supports
Pansy^b^	51	rofecoxib	Completed	No benefit from either support	No further use of supports
*Diclofenac vs paracetamol – trials commencing September 2003 to October 2003*					
Pansy^b^	53	Rofecoxib	Withdrew after 6 weeks – diclofenac side-effect (stomach pains)		Returned to previous treatment
Rosie	31	Paracetamol	Withdrew after 2 weeks – diclofenac side-effect (mouth ulcers)		Returned to previous treatment
Jack	59	no regular medication	Completed	No benefit from either drug	No regular medication (paracetamol when necessary)
George	61	cod liver oil only	Completed	Diclofenac superior	Continued with diclofenac

^a ^All names have been changed to maintain patient anonymity

^b ^One patient undertook their own knee support and drug n-of-1 trial

### Willingness to participate

Interviews began by exploring what had determined each patient's decision to participate. Primarily, all respondents were motivated by the possibility of deriving personal gains such as; pain relief, improved quality of life, and discovery of an effective or more acceptable treatment.

*"If there's anything that I could possibly use that was going to make my quality of life better and I was going to help someone else at the same time, why not? Because I think surgery is very invasive and if you can go without it and still have some reasonable quality of life, why not?" *[Pansy]

*"Well, I says 'well, oh, I'll try that', like, because at the end I'll, I'm going to find out which is the best, either the, what is it, the paracetamol, or the other ones, whichever you're going to give us, at the end I want to find out anyway which is the best ones...so, I'm going to find sommat out at the end of it [laughs]" (Interviewer: "so you think it would be quite helpful for you?") "Yeah." *[George]

Some respondents also gave altruistic reasons for participation:

*"That's why I am happy to try, you know, take part in this trial because you know if it helps, not just if it helps me but if it helps other people, you know, you have to do trials don't you...really it's the only way you find out about things isn't it, it's the only way you go forward is to um by trials and research studies." *[Lyn]

Recruitment to the drug trials was more difficult. Less than half of those approached agreed to participate compared to all those approached about the knee support trial. Some knee support trial participants indicated they would have been less willing to participate had the intervention been pharmacological due to possible side effects or discomfort associated with injections. In contrast, wearing a knee support was perceived as 'unlikely to make things worse' since the intervention did not interfere with any current medical or self-management and presented little inconvenience.

The decision to participate in a drug trial was conditional upon a perception of low risk. Negative views about medicine-taking were mitigated by: knowledge that one could withdraw from the trial at any time; familiarity with the drugs due to previous usage; and the 'n-of-1' trial design, which meant taking tablets for just 2-week periods, since this was perceived to pose little risk of addiction or 'damage to the system'.

*(Interviewer: "You mentioned being worried about getting addicted to them [paracetamol], things like that, so I was quite interested that you actually agreed to be in this study as we're asking you to take paracetamol.") "Yeah but it's not over a length of time, is it? Where, if I was going to take them everyday you do get addicted... this is just a fortnight and change and whatever, so not be too bad... If they [tablets] don't work, if I get a lot of pain, which I haven't got at the moment, I'll just stop it and go back on my normal tablets that I'm taking. Simple as that. Because I ain't going to suffer for nobody." *[George]

Participation was also dependent upon the possibility of personal gain outweighing potential risks. This was illustrated by the accounts of two participants who had exhausted other treatment options and so were 'willing to try anything' to find a 'solution'. The trial offered the hope of improving management of their condition and in one case of avoiding total knee replacement surgery:

*"I just think whether it's because I've had these problems for so many years I just think yes, I'll do anything just to try and kind of find out if something's going to work*." [Rosie]

Reasons for non-participation were not explored in detail since only one refuser (Mary) agreed to an interview. However, Mary's account reinforces the centrality of perceived personal risks and gains to individuals' decisions about participating in an 'n-of-1' trial. Mary was deterred by the risk of adverse drug side effects in case these rendered her unable to fulfil her caring role. At the same time, the trial appeared to offer little prospect of gain since she preferred 'non-medical' self-management of her condition and was satisfied with how she was coping.

*"I do remember reading it and thinking quite hard about it, and talking to my daughters about it...and she said 'I don't think it would be a good idea, Mother' [laughs]...You know, in case I did do one and it did give me problems, you see...and then I wouldn't be able to, um, be well enough myself, I mean this is quite a big house as you can see, and I do all the garden and everything because my husband can't breathe well, he's on oxygen fifteen hours a day so I got to do absolutely everything..." (Interviewer: "Did you think at all that it [the trial] might actually be beneficial to you.") "Er, no I didn't think that at all, no. I was only worried it wouldn't be beneficial [laughs]. That was the bit that worried me more because I manage to cope quite well with the way I do things anyway" (Interviewer: "OK, so you're sort of quite happy as you are?") "At the moment, yeah." *[Mary]

Practical considerations were of importance to participation in both trials. These were individual and dependent upon specific social circumstances, thus demonstrating the importance of offering flexibility to secure participation. For instance, one participant needed home visits as transport was a problem and others required appointments to fit around work commitments.

### Eagerness to complete

The potential for personal gain meant participants were eager to complete their trials. Perseverance was a common sentiment. All patients in the knee support trials experienced problems with their poor fit, pain and discomfort. However, they continued to wear the supports and completed their daily diaries until the end of their trial, with one exception (Mel).

*"That was the only thing that was a bit disappointing [the supports]. I, I sort of hoped that um you know it might be a little bit better. But I persevered and you know I, I kept on doing it you know, for the trial." *[Lyn]

*"Well I mean I said I would do it so I thought, no I'm going to stick with this and I will give it, you know, give it a go...I didn't vary it, she [the nurse] said 7 days [wearing the support]...I've got to admit that I was just thinking oh I've only another day and I can have a bit of relief from this." *[Mel]

Two drug trial participants found it difficult to take so many tablets and had to withdraw due to side-effects (Table 1), however, both were willing to re-start the trial and were disappointed by having to withdraw because this removed their hope of finding an effective treatment.

*(Interviewer: "Will you be disappointed if you're taken out of the trial?") "I don't know, probably not because I wouldn't have to take the tablets but then I would be because I wouldn't know, it would be a bit like well what are you going to try me on now?" (Interviewer: "Are there many of other treatment options for you as far as you know?") "Not at the moment no, I'm seeing this physio but no I don't think there's anything at the moment." *[Rosie]

Daily diaries were completed with very little missing data by all patients and were seen as quick and easy to complete. Only one participant found this tedious.

### Outcomes and added benefits

All patients who completed their trial attended a feedback meeting with their consultant where the results of the diaries were discussed and used to inform a joint decision regarding future treatment (Table 1).

Despite the fact that Pansy and Lyn experienced no treatment benefits, both felt there had been benefits of participating. Lyn gained more insight into her problem, which led her to seek surgical help.

*"Um, I think one of the main things I noted was it, it made me more aware of my problems and aches and pains and it's made me actually decide even though Dr C suggested several times, you know, how do I feel about getting you know, having a knee replacement. And I've been putting it off and putting it off but I'm now feeling as though I really ought to do something about it." *[Lyn]

For Pansy, who experienced side effects during her drug trial and withdrew early, the trial was still informative.

*"I've learned not to mess with my medication again [laughs] any tablets, yeah, I'm not a keen tablet taker anyway, just take them through necessity, um, but it just reinforced problems that I'd had previously which you tend to forget about taking tablets and how long it, you know, it had taken me to sort them out, so in a way it was a relief to get back to taking something that I know was going to have some improvement albeit, not as good as possibly I would like, but um, yes." *[Pansy]

Some respondents found that diary completion provided insight into their condition and helped identify adjustments to daily activities that could be made in order to gain better self-management of symptoms.

*"...it reinforces what actually happened on those days and, and I suppose also again, certain days the pain was at a certain level, and on others [pause] and I could also identify what I'd done to increase that if you know what I mean, so to me, it was helpful in many ways, to know what I should and should not be doing." *[Pansy]

During her knee support trial, Pansy noticed that she seemed to get worse at weekends and was pleased to see this pattern was evident in the diary scores.

*"No that was interesting to see that, 'cause it was only as I started doing that, that it suddenly came to me, well I thought well I'm sure it's getting worse weekends, why? And that's why I identified that, that it's everything that I do during the week I'm doing to extremes at the weekends." *[Pansy]

This additional benefit was also perceived by Amber, who identified that she needed to go for more frequent short walks in order to better control her symptoms.

*"End of every sort of week I sort of go back through it *[the diary] *and look through and you know, Oh yeah I did a bit of extra walking that week and my knee played up but if I didn't do enough walking my knee played up so, I've learnt to sort of do 20 minutes walking now and then sort of stop ...I've learnt a bit out of it." *[Amber]

### Acceptability and understanding of the trial design

In general, participants viewed medical research favourably and regarded it as essential for reaching conclusions about the effectiveness and safety of treatments. Although most had a limited understanding of the 'n-of-1' trial design, several compared it to participation in a conventional randomised trial and understood that they would receive both treatments, which they regarded as more favourable.

Above all, they emphasised the existence of variation between individuals in their responses to treatment, believing that 'everyone is different' (Jack) and that 'what works for some people isn't going to work for others' (George). In this sense, comparing against oneself rather than against others was perceived as more 'logical' and likely to reveal an accurate answer.

*(Interviewer: "She [research nurse] said this would be a trial in which you would be compared with yourself.") "Yes, oh that makes a lot more sense than comparing me, or someone else to me, or me to someone else because we're all different. We're all totally different. Me's me and you's you as it were...that's got to be the way of doing it. Let's say if you get twenty people in a room and they've all got a headache. So you say to them, 'right you can have two paracetamol, and you can have a placebo', maybe half the ones that had the paracetamol it won't make any difference to anyway because they don't affect them so what conclusion can you come to?...because we're all different. Some people can't even take paracetamol because they have a violent reaction to it and I've taken six at a time and even then it hasn't had much affect." *[Jack]

In turn, participants were able to view the trial as 'being about me' (Pansy) and as offering an efficient method of reaching a personalised treatment decision that is tailored to suit individual needs and preferences.

*(Interviewer: "Do you think they [n-of-1 trials] are a good idea?") "Well yes I can say that about this one. I think if it's going to benefit you. It depends what people think but I think this one especially because it was going to benefit me at the end of it to know which tablet was going to be the best one for me. Yes, so yes it just depends what the trial is, doesn't it?" *[Rosie]

*(Interviewer: "Can you see a point in having one and then another [treatment]?") "Well yeah, well its just like anything isn't it? Like with food you eat. You try Cabbage, you don't like that, so you try broccoli. Oh that's quite a funny way of equating it really but you don't know until you've tried anyway do you?" *[Amber]

Two participants (Jack and George) also described the n-of-1 trial as 'fairer' on the basis that each individual would have the opportunity to benefit from an active treatment, while this is not the case in a conventional trial.

*"You could have twenty people in a trial. Ten have a placebo, ten have whatever the tablet is, the one that's going to cure it...there would be that group of people that never got treated for it." (Interviewer: "so does this seem fairer this trial [n-of-1]?") "Of course it is. It just seems odd to me that they say this [n-of-1] is like a new study. I would of thought one would of done that ages ago. I always think it's rather odd and feel sorry for the person that's getting the placebo if they've got a problem because its not doing anything for them." *[Jack]

These factors meant that participation in an n-of-1 trial was likely to yield personal benefit.

## Discussion

This pilot study suggests the acceptability of using n-of-1 trials within secondary care in the UK. Participants viewed the n-of-1 trial as a logical, accurate and 'fair' study design. They recognised and valued the personalised nature of the trial and that it had the potential to offer personal gain, namely improved treatment. Indeed, this possibility meant that individuals were keen to participate and persist with the trial, even where difficulties were encountered. These findings indicate that recruitment to n-of-1 trials is feasible, though barriers were encountered where the intervention was pharmacological due to perceived risks. Further, flexibility (for example, in relation to the time and location of appointments) was identified as important to trial participation and this can be accommodated more easily within the context of an n-of-1 trial design. The findings also suggest that further benefits over and above any gained from the intervention can be derived from involvement in such a study. Some participants gained greater insight into their condition, which improved their self-management. Three patients withdrew before the end of their n-of-1 trial because of intolerance to the interventions. Even for such individuals future treatment is informed from participation in an n-of-1 trial.

Of course, the pilot nature of this study and the sample size involved means that a full exploration of views is unlikely to have been achieved but the findings support and complement other similar research, which has found patient response to be very positive and established evidence of consumer demand for n-of-1 trials outside the UK[5,6,8,9,12]. Nickles *et al *recently published the results of a qualitative study conducted in Australia, where n-of-1 trials are more widely recognised and established[7]. As in this study, Nickles *et al *found that patient participation led to better understanding and management of their condition. The present study has extended this work to the UK where the uptake of such trials has been much slower.

Reported n-of-1 trials have evaluated pharmacological treatments in a range of conditions[5-10,12]. However, few report their use for non-pharmacological treatments within health services. An obvious limitation of the knee support trials, was the lack of blinding and the potential for a patient's pre-conceived preferences and beliefs to bias diary entries. The 'placebo effect' has been estimated to be responsible for up to 30% of many treatment effects observed in conventional randomised trials[17]. In the context of an n-of-1 trial, for some individuals it may be unlikely that prior beliefs can be maintained for several treatment changes if they are not substantiated by real clinical benefits. For others, a placebo effect experienced in the early part of an n-of-1 trial may perpetuate prior beliefs for later treatment periods. If prior preferences and beliefs are maintained for the duration of a trial then there may still be a benefit (albeit non-clinical) from continuing with a specific treatment choice. The impact of an unblinded n-of-1 trial will inevitably depend on both the strength of prior beliefs and the individual. In any case, the presence of multiple treatment periods and structured data collection throughout the study means that the n-of-1 design remains methodologically superior to "trials of therapy" even in the absence of blinding.

The time and cost involved in conducting an n-of-1 trial means that they cannot be performed for all patients for whom a treatment decision is sought. In addition, such trials are only appropriate for patients with chronic and stable conditions and (due to potential carry-over effects from one treatment period to another) for interventions with rapid effect and termination of effect. Due to the presence of multiple treatment periods, outcomes cannot be permanent states such as death. Despite these limitations, when optimal treatment for an individual patient is in doubt and the above criteria are met, the n-of-1 trial offers a methodological improvement to standard clinical practice, essential in the context of evidence-based medicine[4]. Such methods may also improve the doctor-patient relationship by encouraging shared-decision making, which is especially important in the management of chronic disease.

In conclusion, this pilot study suggests that the n-of-1 trial is an acceptable approach to the individualisation of treatment in the UK. Moreover, from the patient's perspective, involvement in such a trial may result in additional benefits. An important next step is to explore the issues raised in this study with a larger and more diverse sample. Further attention should also be directed towards discovering reasons for refusal, especially when considering pharmacological interventions. Similar research should be conducted within primary care in the UK, though it is likely that patient perspectives would be similar to those observed here and by Nikles *et al*[7]. The use of n-of-1 trials in clinical practice is likely to be increased by better understanding of their purpose and potential amongst all stakeholders, not just the patient, and the development of dedicated n-of-1 services.

## List of abbreviations

RCT – Randomised controlled trial

NSAID – Non-steroidal anti-inflammatory drug

OA – Osteoarthritis

MYMOP – Measure your medical outcome profile questionnaire

WOMAC – Western Ontario and McMaster Universities Osteoarthritis Index

## Competing interests

The author(s) declare that they have no competing interests.

## Authors' contributions

All of the authors have been involved in either the conception and design of the research (SB, CP, PD) and/or the analysis and interpretation of the data (SB, CP, LB, GW). All authors have been involved in the drafting of the paper and all have seen and approved the final manuscript.
